# The effect of restrictive compared to liberal intravenous fluid volume on hypotension in adults undergoing major abdominal surgery

**DOI:** 10.1038/s41598-024-65031-2

**Published:** 2024-06-22

**Authors:** Zachary Hollo, Stewart McKenzie, Roman Kluger, Philip Peyton, Andrew Melville, Tuong D. Phan

**Affiliations:** 1 Department of Anaesthesia and Acute Pain Medicine, St Vincent’s Hospital Melbourne, 41 Victoria Parade, Fitzroy, VIC 3065 Australia; 2 Deakin University, 75 Pigdons Road, Waurn Ponds, Geelong, VIC Australia; 3 Bendigo Health, 100 Barnard Street, Bendigo, VIC Australia; 4 Austin Health, 145 Studley Road, Heidelberg, VIC Australia; 5 University of Melbourne, Grattan Street, Parkville, VIC Australia; 6 Alfred Health, 55 Commercial Road, Melbourne, VIC Australia

**Keywords:** Esophageal Doppler, Cardiac output, Fluid therapy, Hypotension, Operative, Blood flow, Predictive markers

## Abstract

In a cardiac output (CO) sub-study of the Restrictive versus Liberal Fluid Therapy in Major Abdominal Surgery (RELIEF) trial, it was shown that restrictive fluid management was associated with lower cardiac index at the end of surgery. However, the association of the fluid protocol with intraoperative blood pressure was less clear. This paper primarily compares rates of hypotension between the two fluid regimens. The haemodynamic effects of these protocols may increase our understanding of perioperative fluid prescription. Using a data set of arterial pressure and cardiac output measurements, this observational cohort study primarily compares intraoperative hypotension rates defined by a mean arterial pressure < 65 mmHg between liberal and restrictive fluid protocols. Secondary analyses explore predictors of invasive mean arterial pressure and doppler-derived cardiac output, including fluid volume regimens and surgical duration. 105 patients had a combined total of 835 haemodynamic data capture events from the beginning to the end of the surgery. Here we report that a restrictive regimen is not associated with a greater proportion of participants who experience at least one episode of hypotension than the liberal regimen 64.1% vs. 61.5% (mean difference 2.6%, 95% CI − 15.9% to 21%, p = 0.78). Duration of surgery was associated with an increased risk of hypotension (OR 1.05, 1 to 1.1, p = 0.038). A fluid restriction protocol compared to liberal fluid administration is not associated with lower blood pressure.

## Introduction

Hypotension is a common and significant physiological stress in patients undergoing major surgery. Intraoperative hypotension is associated with major adverse cardiac, renal and neurological complications in the perioperative period^1–3^. Varying degrees of haemodynamic instability are common during surgery and anaesthesia, yet the duration and severity of hypotension that results in end-organ dysfunction remains unclear. This is reflected by widely heterogenous definitions of intraoperative hypotension in the literature, using varying systolic, diastolic and mean arterial pressure (MAP) thresholds to define hypotension, further impacting the reliability of research endpoints when assessing for the complications of hypotension in the perioperative period^4^.

A recent systematic review of intraoperative hypotension and adverse perioperative outcomes by Wesselink et al. suggests that the risk of acute kidney injury (AKI) and myocardial injury (MI) becomes significant during episodes of hypotension with a MAP < 65 mmHg and gradually increases with both the severity and duration of hypotensive^4^. The reported risk of end-organ injury after non-cardiac surgery from exposures to MAP < 65 mmHg greater than 5 min or any exposure to MAP < 55 mmHg resulted in an increased relative risk or increased odds ratio of between 40 and 100%. The reported risk was even greater with exposures to MAP < 65 mmHg greater than 20 min, MAP < 50 mmHg greater than 5 min, or any exposure to MAP < 40 mmHg. Similarly, a Perioperative Quality Initiative (POQI) consensus statement reported that even brief periods of intraoperative hypotension, reflected by a MAP < 60–70 mmHg, are associated with AKI, MI and death^5^. Consistent with the POQI statement and systematic review, intraoperative hypotension will primarily be defined as a MAP < 65 mmHg throughout this paper.

Intraoperative hypotension defined by relative thresholds of 20% below preoperative MAP or absolute thresholds of MAP < 65 mmHg were both independently associated with postoperative AKI and MI in a retrospective analysis of over 57,000 patients undergoing non-cardiac surgery by Salmasi and colleagues^1^. However, the associations with AKI and MI were no stronger when using relative thresholds compared to absolute thresholds of a MAP < 65 mmHg. This suggests absolute values of MAP < 65 mmHg are comparable to relative thresholds when used to discriminate complications of intraoperative hypotension and offer appropriate clinical haemodynamic targets with the advantage of being a simple single threshold^1,5^.

Perioperative hypotension is most commonly attributed to preoperative fasting and bowel-preparation-induced fluid deficits, anaesthesia-induced vasodilation, extravasation of intravenous fluid and surgery-related blood loss^5,6^. Intraoperative hypotension is often managed by administering intravenous fluid, vasopressor, or both; however, a standardised regimen is yet to be applied in operating theatres^7^. Traditional protocols administering generous volumes of intravenous fluids are postulated to cause overload-related complications such as tissue oedema and weight gain which may lead to worse postoperative outcomes^8,9^. This has resulted in the use of more restrictive fluid regimens which take a zero fluid balance approach in the perioperative period^10,11^.

However, the RELIEF trial, an international randomised controlled trial of almost 3000 high-risk patients undergoing major abdominal surgery showed that there was no difference in disability-free survival up to 12 months after surgery between people receiving restrictive and liberal fluid regimens^12^. Furthermore, a secondary outcome analysis indicated that participants randomised to the restrictive fluid regimen receiving less intravenous fluid were more likely to develop an AKI in the post-operative period^12^. A prospective haemodynamic CO sub-study of the RELIEF trial showed that the liberal group receiving more intravenous fluid had a greater stroke volume and CO at the end of their operation when compared to the restrictive group, which may account for their reduced risk of post-operative AKI^13^. The RELIEF trial protocolized avoidance of hypotension defined by a systolic blood pressure of 90mmHg or an alternative clinician adjusted threshold. Nevertheless, the incidence of clinician-reported hypotension, as defined by an episode of systolic blood pressure < 90 mmHg, was high with a rate of 60.7% in the restrictive and 58.0% in the liberal group (p = 0.13)^12^. The larger RELIEF cohort had inconsistent application of arterial monitoring (62% of cohort) and did not analyse MAP or hypotension events beyond a dichotomous outcome^12^. We seek to examine whether randomisation to a restrictive or liberal fluid regimen impacts the incidence of intraoperative hypotension using continuous arterial monitoring with digital capture, thereby excluding the confounding variable of clinician reporting bias and allowing for an in-depth analysis that includes paired cardiac output measurements. We conducted a post-hoc analysis of the intraoperative data collected in the CO sub-study to address this gap in the literature.

Our primary hypothesis was that randomisation to a restrictive (zero-balance) fluid regimen, receiving less intravenous fluid volume compared to a liberal fluid regimen, would lead to a greater incidence of intraoperative hypotension. Secondarily, we will conduct an exploratory analysis of the variables that are associated with MAP and separately an analysis of variables that are associated with cardiac output.

## Methods

The study was approved by the Alfred Ethics Committee, Melbourne, Australia, approval number: HREC/12/Alfred/58, Pre-RELIEF Sub-study Version 4.1, 19/11/2014. Informed consent was obtained from all participants involved in the study and was conducted in accordance with the ethical principles based on the Declaration of Helsinki. We undertook a post hoc analysis on intraoperative data collected from the CO sub-study of the RELIEF trial, which was registered prior to patient recruitment (ACTRN12615000125527: 11/02/2015). The CO sub-study was a multicentre prospective single-blinded randomised controlled trial of 105 patients undergoing major abdominal surgery who were recruited to the RELIEF trial^13^. Our current study evaluated hypotension as a primary outcome incorporating intraoperative data points, that were collected using digital capture as part of the CO sub-study but have yet to be analysed elsewhere. The incorporation of Oesophageal Doppler derived cardiac index allowed an analysis of the correlation between cardiac output and mean arterial pressure.

In brief, the CO sub-study enrolled adults at greater risk of developing adverse post-operative complications undergoing laparoscopic or open major pelvic and abdominal surgery that included a surgical incision, an expected operative duration of at least 2 h and an expected minimum hospital admission of 3 days. Patients at increased surgical risk included those over 70 years of age or with pre-existing medical comorbidities including but not limited to; morbid obesity, diabetes or pre-existing cardiac or renal disease. Full descriptions of categories determined to be at increased risk are available in the RELIEF trial supplementary index, as these criteria were applied for enrolment in the CO sub-study^12^.

Patients were excluded if they were undergoing emergent surgery, were not expected to survive the perioperative period, were undergoing low-risk surgery (i.e. colostomy closure or laparoscopic cholecystectomy) or had dialysis-dependent end-stage renal failure. In addition, enrolment in the haemodynamic CO sub-study, was also contingent on the trial investigator being proficient in the utilisation of an oesophageal Doppler ultrasound monitor and a patient in whom one could be placed. Patients with known oesophageal disease including varices and those undertaking oesophageal or gastric surgery were thereby excluded. Higher risk patients who were planned for CO monitoring to undertake goal-directed fluid therapy as part of their anaesthetic management were also excluded, as the aim of the pre-RELIEF trial was to observe differences between restrictive and liberal fluid management approaches.

Patients were randomly assigned to either the restrictive or liberal fluid regimen in permuted blocks across three research centres in Melbourne, Australia. Both randomisation groups had predetermined prescriptions of fluid administered upon induction and throughout the operation which are published elsewhere^14^, culminating in patients randomised to the restrictive and the liberal fluid regimens receiving approximately 7.1 ml.kg^−1^ .h^−1^ and 10.7 ml.kg^−1^ .h^−1^ respectively. The fluid protocol stipulated that intraoperative hypotension was treated initially with intravenous fluid in the liberal group, whereas limitations on fluid administration meant that vasopressors were given earlier to treat hypotension in the restrictive group. The CO sub-study protocol was pragmatic and administered by an unblinded anaesthetist who could use appropriate vasopressors for participants in either trial group to achieve a safe target systolic blood pressure, and the agents used were recorded during data collection.

Invasive haemodynamic monitoring was undertaken using an oesophageal Doppler monitor CardioQ-ODM + (Deltex Medical, Chichester, UK) to measure cardiac index, and MAP was measured using an arterial cannula which as a standard is calibrated at the level of the right atrium. While the oesophageal Doppler measurements are continuously available, intermittent probe optimisation is required to obtain the strongest Doppler waveform to enhance the accuracy of the documented CO^15^. The Doppler and paired arterial pressure measurements were recorded every 30 min throughout the intraoperative period and further information on haemodynamic measurement protocols is available in the published CO sub-study^13^. Measurements were limited by the availability of investigators who were present at the beginning and end of surgery, but not always present during the surgery. The number of 30-minutely data capture episodes varied with the duration of surgery and investigator availability. The attending anaesthetist was blinded to the CO parameters collected by the oesophageal Doppler monitor.

### Statistical analysis

Data are presented as median [IQR] or n (%) unless otherwise stated. The primary outcome compares the proportion of patients with at least one episode of intraoperative hypotension in each fluid regimen as defined by a MAP threshold < 65 mmHg. Key secondary blood pressure analyses include assessment of a severe degree of hypotension, as defined by a MAP threshold < 55 mmHg in addition to the number of hypotension events as a proportion of total blood pressure measurements. Hypotension as a binary outcome, MAP < 65, was further analysed with a logistic regression model that included the variable of primary interest, fluid regimen, and also cardiac index, age, sex, body mass index, hypertension and anti-hypertensive medications. Associations with cardiac index were explored with linear regression, mixed effects, in a model that included fluid regimen, MAP, age, sex, body mass index, hypertension and anti-hypertensive medications. Paired CO and MAP readings were included, except outlier readings with a MAP > 200 mmHg and or a CI < 1L/min which would indicate suboptimal Doppler waveforms. A post hoc power calculation shows that 105 patients would have a power of 85% to detect a difference of 30% in the proportion of patients with hypotension (alpha = 0.05). Statistical analysis was undertaken using Stata v17 (Stata Corp. LP, College Station, TX, USA).

## Results

Prior to analysis, 4 patients were excluded from the 109 patients initially recruited due to inadequate data attainment. There were 835 haemodynamic data capture events representing 7.9 and 8.5 events per patient. Presence of hypertension and anti-hypertensive medications are presented in Table 1. Vasopressor type and usage is detailed in Table 2.

**Table 1:** Hypertension and anti-hypertensive medications in patients, grouped by restrictive and liberal fluid regimens, n (%).

	Restrictive (n = 53)	Liberal (n = 52)
Hypertension	32 (61.5)	32 (60)
β-blocker	12 (23.1)	11 (20.8)
Diuretics	12 (23.1)	12 (22.6)
Angiotensin Converting Enzyme Inhibitor(ACEinh)/Angiotensin Receptor Blockade (ARB)	30 (57.7)	24 (45.3)

Hospital: Austin-14, St Vincent’s Melbourne-87, Peter Maccallum-4.

**Table 2:** Intraoperative vasopressor medication use, n (%).

	Restrictive	Liberal
Any vasoactive n (%)	49 (92)	45 (87)
Metaraminol n (%)	28 (53)	28 (54)
Phenylephrine n (%)	8 (15)	19 (37)
Norepineprhine n (%)	3 (6)	2 (4)
Ephedrine/other n (%)	18 (34)	23 (44)

### Primary outcome

**Figure 1:**
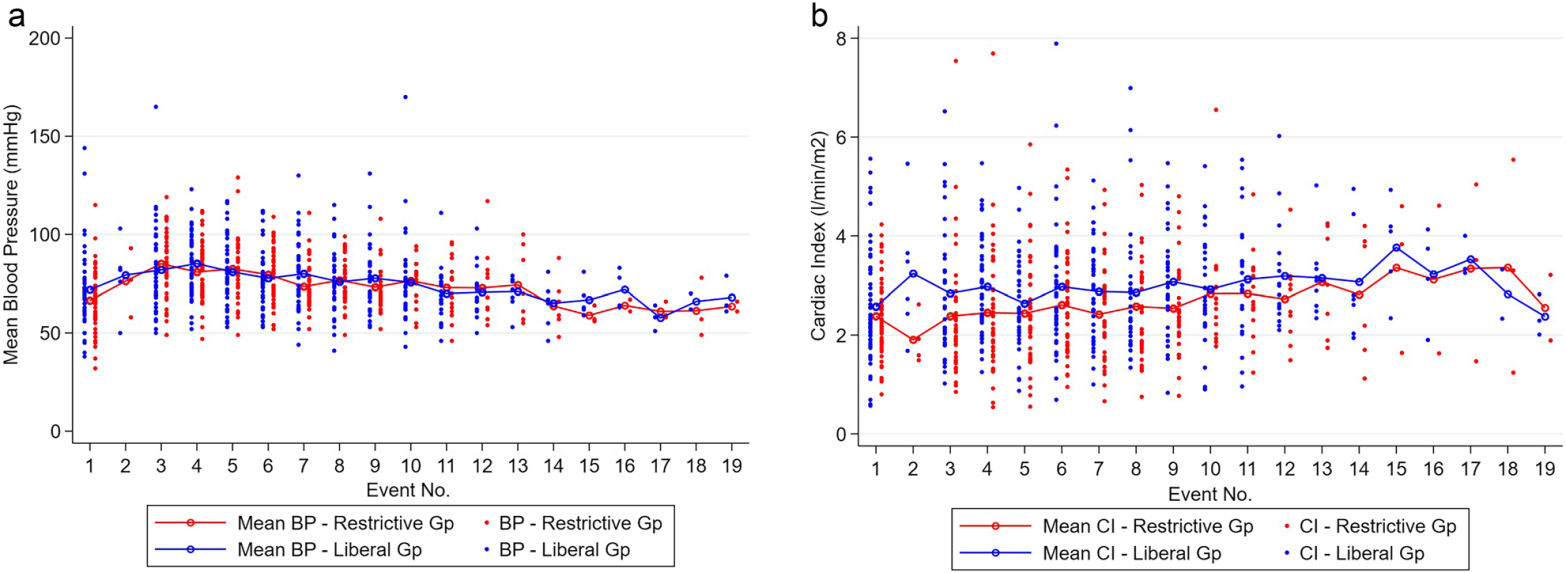
(**a**) Title: Intraoperative Mean Arterial Blood Pressure at 30 min intervals, Restrictive and Liberal Fluid regimens. (**b**) Title: Intraoperative Cardiac Index at 30 min intervals, Restrictive and Liberal Fluid regimens. Episodes represent 30 min intervals of operative duration.

From 105 patients in our study, the restrictive and liberal group had similar proportions of patients with at least 1 hypotension event, 64.1% vs. 61.5% (mean difference 2.6%, 95% CI − 15.9% to 21%, p = 0.78). A plot of mean arterial pressure over time by fluid regimen is presented in Fig. 1a. The number of hypotension events, MAP < 65, was 89 of 405 in the restrictive group compared to 108 of 430 in the liberal group.

### Secondary outcomes

The average of number of hypotension events was not significantly different between the two groups, 0.22 events in the restrictive group compared to 0.25 in the liberal (mean difference 0.03 events, 95% CI − 0.09 to 0.03, p = 0.29). Severe hypotension, MAP < 55 mmHg, had a much lower rate with no difference between the groups, 6% to 7%, p = 0.689. Hypotension was also explored with a mixed effect logistic regression model. The fluid regimen did not significantly change the odds of hypotension events, OR 1.22 (95%CI 0.62 to 2.41, p = 0.567). However, duration of surgery was associated with an increased risk of hypotension, OR 1.05 (95%CI 1 to 1.1, p = 0.038) as shown in Table 3a. Variables that significantly increased cardiac index were randomisation to the liberal fluid regimen (CI 0.44L min^−1^ m^−2^ higher than the restrictive group), duration of surgery (CI of 0. 034L min^−1^ m^−2^ for every 30 min, p < 0.001) and male sex (Table 3b). A plot of cardiac index over time stratified by fluid regimen, see Fig. 1b, shows the effect of fluid regimen and duration of surgery.

**Table 3:** Regression models to predict, (a) Hypotension (b) Cardiac Index.

	OR	95%CI lower	95%CI upper	p-value
(a) Logistic Regression Model: Predictors of Hypotension (MAP < 65)				
Time 30 min intervals	1.053	1.003	1.105	0.038*
Randomisation to Liberal	1.221	0.617	2.414	0.567
Age	1.016	0.985	1.048	0.327
Sex	0.674	0.340	1.337	0.259
BMI	0.985	0.931	1.042	0.597
Hypertension	1.534	0.519	4.540	0.439
Betablockers	0.637	0.267	1.518	0.309
Diuretics	0.610	0.254	1.462	0.268
ACEARB	1.752	0.650	4.720	0.268
(b) Linear Regression Model: Predictors of Cardiac Index				
Time 30 min intervals	1.035	1.019	1.051	< 0.001*
Randomisation to Liberal	1.552	1.163	2.071	0.003*
MAP	1.001	0.997	1.005	0.702
Age	1.004	0.991	1.016	0.554
Sex: M	1.763	1.323	2.351	< 0.001*
BMI	1.000	0.977	1.023	0.996
Hypertension	0.901	0.574	1.416	0.652
Betablockers	0.578	0.399	0.837	0.004*
Diuretics	1.044	0.713	1.527	0.825
ACEinh/ARB	1.270	0.839	1.923	0.258

*p-value < 0.05.

## Discussion

In this post hoc analysis of data collected in the CO sub-study of the-RELIEF trial, we found that a restrictive intravenous fluid regimen was not associated with a greater incidence of intraoperative hypotension when compared to a liberal regimen. The high rates of hypotension, 61.5 to 64.1%, reflect the broad definition which was one or more blood pressure measurements of MAP < 65mmHg.The proportion of hypotension events as a total of all events was reassuringly lower 21.9 to 25.1%, however it highlights the common occurrence of mild hypotension in this cohort, despite the ubiquitous use of vasoactive medications in both groups. The proportion of patients in the restrictive and liberal groups who received any vasopressor medication intraoperatively was very high and similar between the fluid regimens (92% vs 86%, p = 0.32)^13^. A threshold of severe hypotension, MAP < 55 mmHg, had a much lower inidence of hypotension events 6% and 7% in the restrictive and liberal groups.

The restrictive fluid protocol aimed to minimise perioperative fluid administration with a goal of “zero balance” post-operatively. To achieve this, the restrictive group received a lower maintenance intravenous fluid rate and only received boluses of intravenous fluid when hypovolaemia was identified clinically. In the absence of clinical identification of hypovolaemia, the restrictive group was protocolised to receive vasopressor to treat hypotension in the first instance. This approach resulted in no difference in the proportion of patients with hypotension in the restrictive fluid group when compared to the liberal fluid regimen. Similarly, a liberal fluid regimen which generally encourages fluid bolus administration for hypotension as the initial response was not protective in reducing the incidence of hypotension in a randomised perioperative fluid trial by Joosten et al.^16^. Table 4 compares hypotension rates among intraoperative haemodynamic research, showing a high proportion of patients with at least a single reading of hypotension when defined using a similar definition^7,17^. The table demonstrates a large variation in the methodology of these studies, including inconsistent measuring and reporting of cardiac output.

**Table 4:** Comparison of studies reporting hypotension, ns = not statistically significant.

Study: author/year/type of surgery	No. patients	Rate of hypotension (Spot measure)	Threshold	Analysis of hypotension with a minimum time period	Total no.of measurements	Time interval between measurements	Cardiac output measured(Doppler, Arterial Waveform)/%change	Fluid volume measured	Prospective or retrospective data capture	Arterial line or NIBP	Method of capture: Digital/Manual
Proportion of patients with a hypotensive event											
Hollo et al. 2024 (Major abdominal)	105	Restrictive fluid volume 64.1%, Liberal 61.5%^ns^	MAP < 65	N	835	30 min	Y (Doppler)/inc 15%	Y	Pros	Art	Digital
Wickham, 2021 (All non-cardiac)	4750	61%	MAP < 65	Y	N/A	N/A	N	N	Pros	NIBP	Manual
Futier, 2017 (Major non-cardiac)	145	84.80%	SBP < 80 or decrease < 40%	N	3028	10 min	Y (Arterial)/inc 22%	Y	Pros	Pros	Digital
Ariyarathna 2022 (Non-cardiac)	830	25.9% SBP < 90, 11.9% MAP < 60	SBP < 90 or MAP < 60	Y	N/A	5 min	N	N	Retro	NIBP	Manual
Proportion of hypotensive events											
Hollo et al. 2024	105	Res 21.9, Lib 25.1%^ns^	MAP < 65	N	835	30 min	Y (Doppler)/inc 15%	Y	Pros	Art	Digital
Kouz, 2022 (Major abdominal)	100	35.10%	MAP < 65 or MAP 66–75 + norad	N	1798	15 min	Y (Arterial)	Y	Pros	Pros	Digital

The absence of a beneficial effect of liberal fluid therapy in reducing hypotension rates, supports observations that intraoperative hypotension may be secondary to anaesthesia-induced vasodilation initially and later surgical duration; reinforcing recommendations that the first step in management of intraoperative hypotension when perioperative assessment suggests the patient is euvolaemic, should be to treat the suspected reduction in systemic vascular resistance with vasoactive agents^18^. Futier et al. demonstrated that using vasopressors to target tight intraoperative and post-operative arterial pressure control can reduce the risk of perioperative organ injury in a trial of 292 patients at high-risk of post-operative complications undergoing major abdominal surgery^7^.

Beyond analysis of pressure targets, analysis of CO showed it increased with the duration of surgery in both groups. This increase is likely a reflection of demand driven changes in CO. The stress of surgery is known to induce a greater demand for oxygen delivery^19^, which is met with increases in CO in both trial arms, however this effect is more pronounced in patients randomised to the liberal fluid regimen^13^. Further analysis in this study shows that in a model of 10 variables, cardiac index in the liberal group was 0.44L min^-1^ m^-2^ higher than the restrictive group. Conversely, MAP trended down with surgical duration. This decrease in MAP coupled with an increase in the cardiac output in both fluid regimens suggest a decrease in systemic vascular resistance with surgical duration. Clinical management of hypotension should therefore take into consideration surgical duration and may support the initiation of vasopressor for longer procedures. Furthermore, the greater CO output in the liberal group is not matched by fewer hypotension events than the restrictive regimen. However, systemic vascular resistance was not directly measured in our study, limiting conclusions pertaining to differences in vasomotor tone between the two fluid regimens. Our study showed that invasive MAP measurements were not concordant with Doppler-derived cardiac output values, which has previously been described in patients in ICU with sepsis^20^. A study of 100 patients undergoing major abdominal surgery by Kouz et al. further supported the notion that there is no clinically meaningful relationship between MAP and CO, which were measured using an arterial cannula^21^.

There was also a finding of a gender difference with the male sex associated with a higher cardiac output. This has a physiological basis with a review by Pierre et al. on sex differences in cardiovascular variables summarizing that women have a smaller cardiac output; a difference that allometric scaling by lean body mass reduces but does not eliminate^22^. Preoperative B-blocker use was understandably associated with a significant decrease in cardiac index from an anticipated reduction in heart rate.

The strength of our analysis is it contains a cohort that included patients randomised to two fluid volume regimens and incorporated Doppler derived cardiac output which increases our ability to draw conclusions about potential causes of hypotension. Attributing an episode of hypotension to reduced systemic vascular resistance is possible when the cardiac index is observed to have increased. Similarly, measurement of the cardiac output has allowed us to observe increased cardiac output in the liberal group independent of the MAP.

Limitations of the study are primarily that MAPs were measured at single timepoints with the threshold of MAP < 65 mmHg representing mostly mild episodes of hypotension which may be of short duration. This study does not attempt to analyse severe or prolonged episodes of hypotension. While longer periods of intraoperative hypotension are known to have the greatest increase in the risk of post-operative complications, short periods are also associated with increased risk^2,4,5,17^. Blinding of the fluid regimen in the RELIEF study was not attempted given the pragmatic study design^12^. Bias in the haemodynamic measurements was minimised by utilising digital capture of the Doppler and pressure variables and concealment of the group allocation until the primary statistical analysis was undertaken.

Furthermore, the relatively small subgroup of patients we studied reduces the ability of the study to detect differences smaller than 30% of the proportion of patients with hypotension. We are also unable to explore the effect of vasoactive agents beyond the number of patients that had vasoactive support and type of vasoactive medication used; a difference in the vasoactive drug dosage may confound the findings of the study. Future intraoperative hypotension research should appraise vasopressor dosing regimens, in addition to duration of hypotension to identify periods of sustained hypotension which may be more closely associated with adverse outcomes.

The large multicentre RELIEF trial found that intravenous fluid restriction was associated with increased risk of post-operative acute kidney injury, and the CO sub-study showed that fluid restriction was also associated with lower cardiac index than the liberal regimen^13^. This is not reflected in differences in the incidence of intraoperative hypotension in our retrospective analysis. Optimising intraoperative fluid balance and CO may help to maintain renal blood flow. This offers a plausible physiological mechanism of mitigating pre-renal acute kidney injury in high-risk patients undergoing major abdominal surgery, however additional research is required to establish whether CO optimisation strategies reduce the incidence of perioperative kidney injury.

In conclusion, the restrictive fluid regimen in our study was not associated with a greater proportion of hypotensive events than the liberal regimen. Duration of surgery was associated with a decrease in blood pressure even while the cardiac output rises. The divergent response indicates a decrease in the systemic vascular resistance over time. Understanding these haemodynamic associations may aid clinician decisions to achieve the twin haemodynamic aims of a safe blood pressure and an optimised cardiac output.

## Data Availability

The datasets generated and/or analysed during the current study are not publicly available due to data confidentiality and not prespecified during the consent process but are available from the corresponding author on reasonable request.
